# Effect of two-weeks of school-based sprint training on physical fitness, risk factors for cardiometabolic diseases and cognitive function in adolescent girls: A randomized controlled pilot trial

**DOI:** 10.3389/fspor.2022.884051

**Published:** 2022-08-05

**Authors:** Ryan A. Williams, Karah J. Dring, John G. Morris, Caroline Sunderland, Mary E. Nevill, Simon B. Cooper

**Affiliations:** Sport Health and Performance Enhancement (SHAPE) Research Group, Department of Sport Science, Nottingham Trent University, Nottingham, United Kingdom

**Keywords:** cardiometabolic health, cognitive function, exercise training, adolescence, physical activity

## Abstract

**Background:**

School-based physical activity interventions are accessible to most adolescents and could enhance adolescent cardiometabolic health and cognition; yet the feasibility and success of school-based physical activity interventions is understudied.

**Methods:**

Sixteen adolescent girls (age: 11.7 ± 0.3 y; height: 1.58 ± 0.07 m; body mass: 45.5 ± 9.2 kg) were randomized to either an intervention (2-weeks sprint training; *n* = 8) or control group (continuation of regular physical activity levels; *n* = 8). Following familiarization, all participants completed baseline measurements including fasted and postprandial capillary blood samples, a battery of cognitive function tests (Stroop Test, Sternberg Paradigm and Flanker Task), and an assessment of physical fitness (20 m sprint and multi-stage fitness test). The intervention group completed 2-weeks progressive sprint training (3 sessions per week: week one 6 × 10 s sprints, week two 8 × 10 s sprints). Follow-up measurements were completed 48 h after the final sprint training session. Data were analyzed *via* ANCOVA to examine between group differences at follow-up whilst controlling for baseline score.

**Results:**

Accuracy in the intervention group during the three-item Sternberg paradigm was greater when compared with the control group (Intervention: 99.6 ± 1.1%; Control: 97.7 ± 2.2%, p = 0.046). BDNF concentration was also higher in the intervention group at follow-up than control group (Intervention: 39.12 ± 9.88 ng.ml^−1^; Control: 22.95 ± 9.13 ng.ml^−1^, p < 0.001). There were no differences at follow-up between the intervention and control group for measures of cardiometabolic health (fasted cytokine concentrations or postprandial glycaemic and insulinaemic responses) or on the Stroop Test or Flanker Task (all p > 0.05). However, the intervention group reported enjoying the sprint training and that they found the sessions valuable.

**Conclusion:**

Two-weeks sprint interval training in a school-setting enhanced working memory and increased concentrations of BDNF in adolescent girls. The intervention was deemed enjoyable and worthwhile by the adolescent girls and thus the longer-term implementation of such an intervention should be examined.

## Introduction

Engagement with physical activity during adolescence is associated with enhanced physical fitness (Rowlands et al., 1999), cardiometabolic health (Dring et al., 2019), cognitive function (Vanhelst et al., 2016), and academic achievement (García-Hermoso and Marina, 2017). Despite the well documented benefits of physical activity for adolescents, it is alarming that such a small proportion of boys (24%) and girls (18%), aged 5 to 15 years in the UK, meet the recommended physical activity guidelines of 60 min of moderate-to-vigorous physical activity per day (Scholes and Mindell, 2016). Of additional concern is that adolescent physical activity levels decline throughout puberty, with an average 7% reduction in physical activity each year from age 12 to 19 years (Dumith et al., 2011).

School-based physical activity interventions have become of interest, and if successful in improving physical fitness, cardiometabolic health and cognition, have the potential to reach virtually all adolescents. However, the effect of school-based physical activity interventions on the cardiometabolic health of adolescents has been discrepant. For example, 4 weeks of sprint training (3 running sessions per week, 5–6 × 30 s) improved predicted VO_2peak_ (Δ 5.03 ml·kg·min^−1^) and reduced a clustered risk score for cardiometabolic disease (waist circumference, systolic blood pressure, fasting glucose, HDL-c, and triglycerides; Δ −0.22 AU) (Martin-Smith et al., 2019). On the other hand, 2-weeks of cycling at 90% peak power output (3 sessions per week, 8–10 × 1 min with 75 s rest) had no effect on traditional risk factors such as fasting glucose (Pre: 5.05 ± 0.3 vs. Post: 5.00 ± 0.3 mmol·L^−1^) and insulin (Pre: 19.41 ± 8.4 vs. Post: 18.63 ± 3.5 μU·ml) concentrations (Cockcroft et al., 2019) or postprandial markers of metabolism (Glucose tAUC: Pre; 24.56 ± 1.49 vs. Post; 24.74 ± 1.48 mmol·L^−1^·4.25 h. Insulin tAUC: Pre; 223 ± 51 vs. Post; 228 ± 51 mmol·L^−1^·4.25 h) (Bond et al., 2015). Although the aforementioned work was considered high-intensity in nature, the exercise stimuli involved different modalities and exercise volumes. Furthermore, it could be argued that a cycling modality is not necessarily ecologically valid in a school-setting; with running easier to implement in schools given the lack of equipment and facilities required. Finally, the two shorter duration studies (Bond et al., 2015; Cockcroft et al., 2019) did not enroll a control group, which is a required element of intervention studies to determine treatment effects.

Markers of chronic low-grade inflammation, such as cytokines—particularly interleukins (IL) -are associated with cardiometabolic risk, which is suggested to manifest in childhood and adolescence (Balagopal et al., 2011). The prototypical cytokine, IL-6, is involved in the development of visceral fat-mediated insulin resistance (Shuster et al., 2012). However, the focus on these outcomes following exercise interventions in youth is sparse (Buchan et al., 2013; Logan et al., 2016). Eight weeks of circuit-based exercise (Logan et al., 2016) or 7 weeks of running (Buchan et al., 2013) found no change in IL-6 concentrations, though these studies were all conducted in older adolescents (16–17 y) with no information on younger adolescents to date. Furthermore, other cytokines (such as IL-10, IL-1β and IL-15) are of interest (Gleeson et al., 2011) but have not been examined in this population.

Exercise interventions implemented to improve cognitive function in adolescents have received less attention (Wassenaar et al., 2020). Of the available evidence to date, most school-based interventions are between 6 and 12 weeks and typically use moderate intensity exercise (60–70% HR_max_), with interventions focusing on coordinative exercise (Ludyga et al., 2018), team games (Schmidt et al., 2015) or circuit-based exercise (Chen et al., 2016). These interventions have led to lower error rates during a shifting task (Schmidt et al., 2015; Chen et al., 2016) and improved response times during congruent and incongruent levels of a Stroop task (Ludyga et al., 2018). Whilst these study findings promote longer-duration-moderate intensity exercise to improve cognitive function, they lack ecological validity and would not feasibly be introduced as part of the school day due to their time-consuming nature (typically lasting ~ 40 min per session). To tackle this, school-based physical activity—outside of scheduled PE lessons—might benefit from a shorter duration (that could be conducted during break times/morning form) (Boyle et al., 2008; Bond et al., 2017). As a result of being shorter in duration, there would be scope to focus on higher intensity activity. Only one study to date has examined the effect of high-intensity interval training (HIIT) [combined aerobic and resistance exercise training (~75% HRmax)] over a period of 8 weeks on cognition and reported small-moderate improvements in executive function performance (Costigan et al., 2016).

A suggested mechanism for some of the cognitive improvements seen following exercise interventions is through the secretion of brain-derived neurotrophic factor (BDNF); which plays an important role in neuroplasticity and memory (Piepmeier and Etnier, 2015). Only one study to date has investigated the effects of an exercise intervention on BDNF concentration in adolescents (Jeon and Ha, 2017). A dose-response increase in BDNF concentration was found following 12 weeks of treadmill running, with the highest intensity group (70% V°O_2max_) seeing the greatest increase and a concomitant improvement in working memory. It is therefore imperative that more data are collected regarding BDNF changes in response to exercise training that may be related to exercise-induced improvements in cognitive function in adolescents.

With regards the ecological validity of the aforementioned exercise interventions for the improvement of cardiometabolic health (Bond et al., 2015; Cockcroft et al., 2019) and cognition (Schmidt et al., 2015; Chen et al., 2016; Ludyga et al., 2018), there has been little consideration for the adoption of exercise within the school day. Of note, is that any school-based physical activity intervention should require minimal equipment so that set up time is reduced, the intervention is accessible to all adolescents across the school and so that equipment costs are not a barrier preventing schools from engaging with the intervention. However, previous research has required cycle ergometers (Bond et al., 2015; Cockcroft et al., 2019) or specialist equipment for the circuit-based exercises (Schmidt et al., 2015; Chen et al., 2016), which each have associated costs, increased set-up times and require specialist knowledge from the teacher supervising the session. Furthermore, time constraints have been highlighted as a major barrier to the attainment of physical activity at school (Boyle et al., 2008), therefore targeting shorter duration exercise sessions might be more suitable. Therefore, future research should consider the simplicity, and the time commitment required, of the school-based physical activity intervention to ensure it addresses any potential barriers to whole-school inclusion.

Finally, girls are typically less physically active than boys during adolescence (Dumith et al., 2011; Scholes and Mindell, 2016). Considering this and given that physical activity levels decline throughout adolescence, especially in females (Dumith et al., 2011), targeting younger, female adolescents for exercise interventions provides an opportunity to attenuate this decline. Furthermore, by focusing a school-based intervention on the needs of a specific population, such as adolescent girls, increases the likelihood of the intervention being successfully implemented thus resulting in sustained behavior change.

Therefore, the aims of the present study were to investigate the effects, and the perceived enjoyment, of a 2-week, school-based sprint-interval training intervention on physical fitness, risk factors related to cardiometabolic disease, and cognitive function, in adolescent girls.

## Materials and methods

### Experimental design

**Figure 1:**
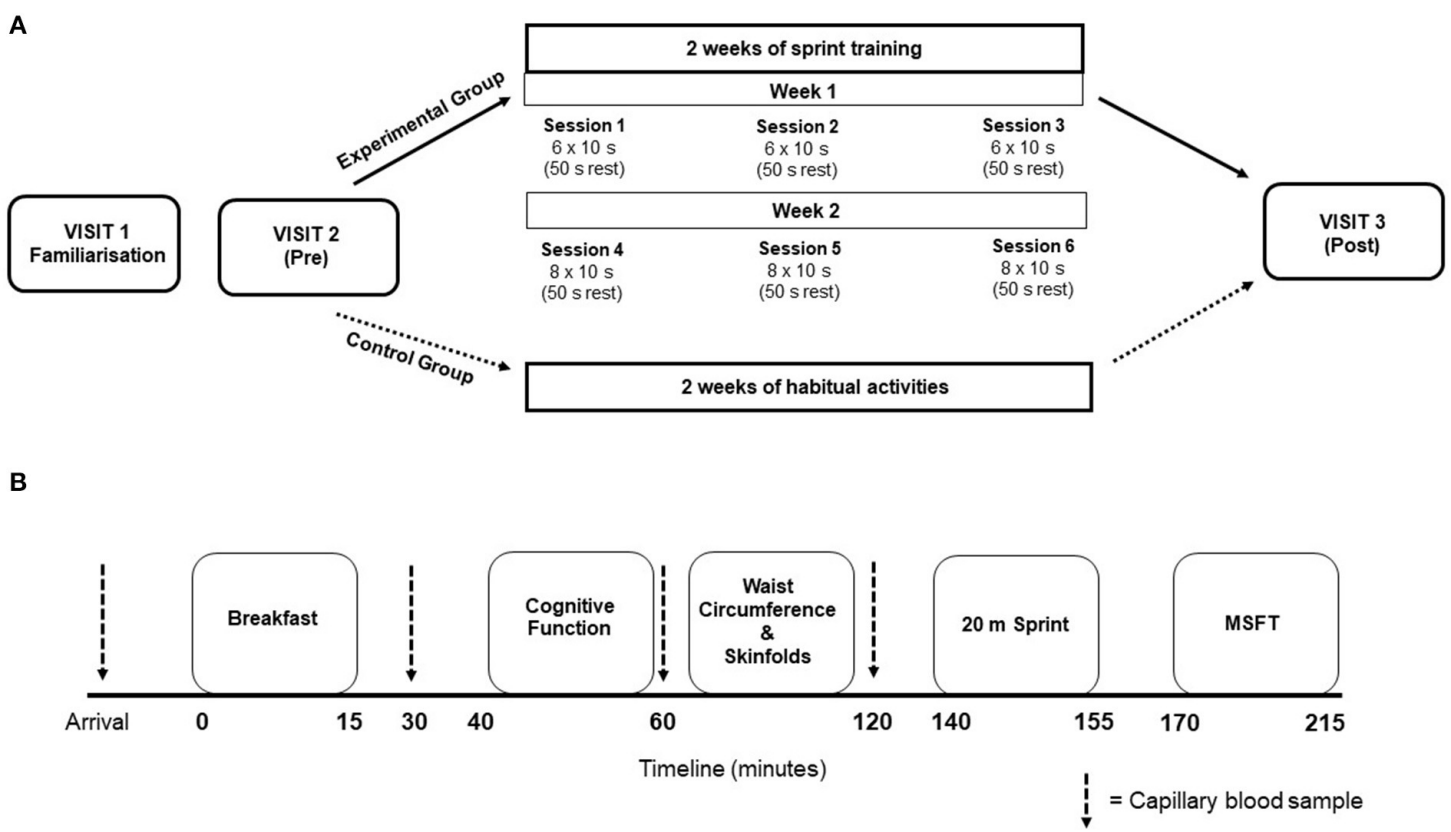
**(A)** Schematic representation of the overall study design. Training session details indicate the number of sprints performed. **(B)** Visual representation for the timeline of measurements during experimental visit's 2 and 3. Thick dashed lines represent the timing of capillary blood samples.

The study conformed to the Declaration of Helsinki guidelines and was approved by the Institutional Human Ethics Committee. Participants were randomized to either the intervention group (sprint training) or a control group (continued their daily routines)—this was achieved by the liaising teacher selecting participants names from a container and using the “ABBA” approach for group assignment. The study consisted of three laboratory-style visits in a school classroom and six training sessions (over 2 weeks) at the school facilities (for the intervention group only), which took place over a 4-week period (Figure 1A). The first visit was an initial familiarization. The second visit consisted of the pre-intervention measures, which took place 48 h prior to the training period. The experimental group then completed six supervised sprint sessions over the subsequent 2 weeks, whereas the control group continued their normal daily routines. All participants completed the third experimental visit consisting of the post-intervention measures, which took place 48 h after the final training session (or at an equivalent time in the control group). To avoid the self-selection of active individuals, and to reward participants for their time and effort, all participants received a gift voucher (Amazon) upon completion of the study.

### Participants

A local secondary school was approached and advertised the study to several classes in year 7, whereby participants were asked to register their interest. Twenty adolescent girls were registered their interest and were given a study documentation pack. The school was from an area in England in the 10^th^ decile on the Index of Multiple Deprivation (least deprived decile). Written parental consent and child assent were obtained prior to enrolment in the study. A health screen was completed on behalf of the participant by the parent/guardian, which was checked by a member of the research team to ensure there were no medical conditions that would affect participation in the study. This included existing neurological and/or underlying health conditions, such as family history of cardiovascular disease or diabetes. All participants initially enrolled satisfied these criteria. Groups of participants from two different classes were randomly allocated to the intervention and control group (n = 10 in each group). Four participants were not able to complete the study due to illness (n = 2) or isolation due to COVID-19 (*n* = 2). This resulted in a total of 16 participants (8 in each group) completing the study. A summary of baseline participant characteristics can be seen in Table 1.

**Table 1:** Descriptive summary of baseline participant characteristics for the intervention and control groups.

	**Group**			
	**Intervention (*****n*** = **8)**		**Control (*****n*** = **8)**	
**Variable**	**Mean ±SD**	**Range**	**Mean ±SD**	**Range**
Age (y)	11.8 ± 0.2	11.6–12.0	11.6 ± 0.4	11.2–12.2
Height (cm)	155.3 ± 7.4	142.5–165.5	159.7 ± 7.4	147.9–171.7
Maturity offset (y)	0.01 ± 0.32	−0.49–0.42	0.13 ± 0.52	−0.59–1.14
Body mass (kg)	43.2 ± 8.6	33.8–57.4	47.6 ± 9.9	33.8–60.5
BMI (kg·m^−2^)	17.8 ± 2.2	15.0–21.6	18.5 ± 3.0	15.3–23.2
BMI centile	44.4 ± 31.7	5.3–89.4	52.9 ± 36.0	9.3–94.9
Sum of skinfolds (mm)	50.0 ± 17.3	30.2–83.5	58.6 ± 30.0	23.4–117.5
Waist circumference (cm)	61.8 ± 5.3	56.9–72.7	64.8 ± 6.2	57.0–74.7

SD, Standard Deviation; BMI, Body Mass Index.

### Experimental visits

#### Visit one: Familiarization

During the familiarization participants underwent anthropometric measurements of height (cm), sitting height (cm) and body mass (kg). Participants were given a full practice of the cognitive function test battery, as well as being familiarized with a capillary blood sample. The experimental group also practiced the sprint protocol to be used during the training sessions.

#### Visits two and three: Pre- and post-intervention testing measures

Participants avoided strenuous physical activity and recorded their dietary intake 24 h prior to the main experimental visits; dietary intake prior to the pre-intervention measures was replicated prior to the post-intervention measures. Participants attended the classroom where the laboratory was set up at ~ 08:30 am after a 12 h overnight fast, with *ad libitum* water consumption allowed at all times. Participants provided a fasted capillary blood sample, after which they were provided with a standardized breakfast. Postprandial capillary blood samples were taken at 30-, 60- and 120-min after breakfast for the assessment of blood glucose and plasma insulin concentrations. Participants completed the cognitive function test battery 45 min post-breakfast. Participants also underwent waist circumference and skinfold thickness measurements. The physical fitness test battery was completed ~20 min following the final blood sample, with the 20 m sprint test completed first, followed by the multi-stage fitness test after a 10 min rest (Figure 1B).

#### Sprint training sessions

Participants in the experimental group completed a two-week sprint-based training programme. The training sessions took place on an outdoor court at the school, with 3 sessions scheduled per week (Monday, Wednesday, and Friday). The sessions were conducted during morning form time (08:45–09:15), so that it did not interfere with the school day or detract from the participant's usual Physical Education lessons. Upon arrival to the classroom, participants were fitted with a heart rate monitor (Firstbeat Technologies Ltd, Finland) and a global positioning (GPS) unit (Catapult Sports, Melbourne, Australia) to monitor the internal and external load of the sessions. Each session started with a brief warm up, consisting of 2 shuttles at a self-selected pace. The training sessions consisted of 10 s maximum effort sprints, with 50 s of passive (walking) recovery, performed as shuttles between two cones which were placed 40 m apart. Participants were encouraged to cover as much distance as possible within the 10 s. A custom audio file was used to administer the sprint session, with a “bleep” signaling the start and end of the sprint, as well as audio notifications half-way through the rest period and 5 s before the start of the next sprint. The cool down consisted of 2 min walking. The first 3 training sessions consisted of 6 × 10 s sprints (6 min per session, weekly total of 18 min). The final 3 sessions consisted of 8 × 10 s sprints (8 min per session, weekly total of 24 min). The total training time over the intervention was 42 min (60 min including warm up and cool downs). Participants were given verbal encouragement during each sprint.

### Experimental procedures and measurements

#### Anthropometric and body composition measurements

Stature, accurate to 0.1 cm, was measured using a Leicester Height Measure (Seca Ltd, Birmingham, UK) and body mass, accurate to 0.1 kg, was measured using a Seca 770 digital scale (Seca Ltd). Sitting height (cm) was also measured and this was used, along with height, to estimated maturity offset (as a marker of somatic maturity), using previously described methods (Moore et al., 2015). Height and body mass were used to calculate body mass index (kg·m^−2^). The sum of four skinfold sites were measured to the nearest 0.1 mm (triceps, subscapular, supraspinale and front thigh). All measurements were conducted by a trained kinanthropometrist, twice in rotation and on the right side of the body. An average of the two measurements was used, unless the difference between the two was ≥ 5%, in which case a third measurement was taken and the median value used. Waist circumference was measured to the nearest 0.1 cm.

#### Standardized breakfast

An ecologically valid breakfast was used as a test meal to assess postprandial responses, which has been used successfully before in this population (Dring et al., 2019, 2020; Williams et al., 2021a,b). The breakfast was standardized to provide 1.5 g·kg^−1^ body mass of carbohydrate and consisted of; cornflakes, milk, white toast and butter. Participants were given 15 min to consume the meal. If any leftovers remained during visit 2, these were weighed and subtracted from the breakfast consumed on visit three to ensure identical meal provision for pre- and post-measurements.

#### Capillary blood samples

A fasted capillary blood sample was taken for the determination of a range of pro- and anti-inflammatory cytokines (IL-6, IL-1β, IL10 & IL-15), serum brain-derived neurotrophic factor (BDNF) as well as plasma insulin and blood glucose concentrations. Three additional samples were collected at 30-, 60- and 120-min post-breakfast for determination of plasma insulin and blood glucose concentrations.

To increase capillary blood flow, participants' hands were warmed *via* submersion in warm water prior to sample collection. Blood was collected into 300 μl EDTA coated microvettes (Sarstedt Ltd, UK) for separation into plasma. A single whole blood sample was collected using a pre-calibrated glass pipette (Hawksley Ltd, UK) and immediately deproteinised in 250 μl ice-cooled perchloric acid (2.5%). These samples were centrifuged at 1,000 g for 4 min, at 4°C (Eppendorph 5415C, Hamburg, Germany). Additional blood was collected into a 300 μl microvette, coated in clotting activator, for the separation of serum. The sample was allowed to rest for 30 min at room temperature, before centrifugation at 10,000 × g for 5 min. All samples were frozen immediately at −20°C and transferred to −80°C as soon as possible (typically within ~ 5 h).

#### Blood analyses

Blood glucose concentrations were determined in duplicate using a commercially available assay (GOD/PAP method, GL364, Randox, Ireland). Plasma insulin concentrations were determined using a commercially available ELISA, in line with the manufacturer's instructions (ELISA; Mercodia Ltd, Sweden). Blood glucose and plasma insulin total area under the curve (tAUC) following the standardized breakfast were calculated (GraphPad Prism 7, GraphPad Software, USA), using methods described previously (Wolever et al., 1991). Homeostatic Model Assessment of Insulin Resistance (HOMA-IR) was calculated as an index of insulin resistance (Matthews et al., 1985). Plasma IL-6,−10,−15 and 1β concentrations were determined simultaneously with an automated SimplePlex immunoassay, using the anti-body-based Ella system (ProteinSimple, BioTechne, Oxford, UK), per the manufacturer's instructions (Aldo et al., 2016). All analytes were measured in triplicate and concentrations were determined using preloaded calibration curves. Serum BDNF concentrations were determined using commercially available methods, in accordance with the manufacturer's instructions (Quantikine ELISA, R & D Systems Europe Ltd, UK).

#### Cognitive function test battery

The cognitive function test battery lasted approximately 12 min and consisted of the Stroop test, Sternberg paradigm and the Flanker task. All tests were completed on a laptop computer (Lenovo ThinkPad T450; Lenovo, Hong Kong). Each test, and test level, were preceded by practice stimuli to re-familiarize participants (the data for these practice stimuli were discarded). The tests were conducted in silence and in a darkened room with noise-canceling headphones to minimize external disturbances.

The Stroop test consists of two levels, congruent and incongruent, assessing selective attention and executive function (specifically the domain of inhibitory control), respectively (Miyake et al., 2000). The Sternberg paradigm measures working memory (Sternberg, 1969) and consists of three levels of ascending difficulty (one-, three- and five-item). The Flanker task consists of two levels (congruent and incongruent) and assesses attention and inhibitory control (Eriksen and Eriksen, 1974). Full details of the cognitive testing battery are reported elsewhere (Williams et al., 2020). For each test, the outcome variables of interest were the response time of correct responses [ms] and percentage of correct responses made.

#### Assessment of physical fitness

##### Cardiorespiratory fitness (multi-stage fitness test)

Cardiorespiratory fitness was assessed using the multi-stage fitness test (MSFT) (Ramsbottom et al., 1988). The test requires participants to complete progressive shuttle runs (20 m) until volitional exhaustion, or until they are unable to maintain the designated speed. The test beings at a speed of 8.0 km·h^−1^ (level one), which increases to 9.0 km·h^−1^ (level two) and then increases by 0.5 km·h^−1^ for each subsequent level. Participants were “paced” by a member of the research team. Verbal encouragement was provided by the research team and heart rate (Firstbeat Technologies Ltd, Finland) was monitored continuously throughout to ensure maximum effort. Performance on the test was determined by the total distance covered (m).

##### Twenty-meter sprint

Participants completed maximum effort 20 m sprints for the assessment of sprint speed performance. After a warmup, participants had a practice attempt at the sprint. Following this, participants completed a minimum of three maximum effort sprints (separated by ~ 1 min). Participants completed up to two additional sprints if their final sprint was their fastest. Sprint times were recorded using infrared timing gates (Brower Timing Systems IRD-T173, Draper, UT, USA). The criterion measure was the fastest 20 m sprint time for each participant.

#### Perceived enjoyment of sprint sessions

The enjoyment of the training sessions was assessed using a revised version of the Physical Activity Enjoyment Scale (PACES), which has been adapted and validated for adolescent girls (Motl et al., 2001). Briefly, the scale consists of 16 statements which originally begin with “When I am physically active…,” which were adapted to “When I am taking part in the sprint sessions…,” followed by the statement (e.g., “I enjoy it”). Items were rated on a 5-point Likert scale (1 = “disagree a lot” to 5 = “agree a lot”). Total activity enjoyment was calculated by summing the 16 responses (seven of which were reverse scored), resulting in a possible range of 16–80, with higher scores reflecting higher enjoyment. The PACES was completed after the first (6 × 10 s sprints) and fourth (8 × 10 s sprints) training sessions, within 5 min of finishing the exercise.

#### Training load monitoring

##### Heart rate (internal load)

Heart rate was measured continuously throughout each training session (Firstbeat Team Sport System, Firstbeat Technologies Ltd, Finland) to provide internal load characteristics. Average and maximum heart rate were extracted from each training session. The maximum heart rate (HR_max_) achieved during the multi-stage fitness test was used to calculate the average and maximum relative exercise intensity (%HR_max_) of each session.

##### Global positioning system (GPS) (external load)

GPS was used to quantify the external workload of each training session using PlayerTek units (Catapult Sports, Melbourne, Australia). The units were fitted to sit between the scapulae, using an elasticated shoulder harness. After each training session, the data were downloaded to the PlayerTek software. The variables of interest were top speed (m·s^−1^) and total distance covered across all 10 s sprints (m).

### Statistical analyses

Statistical analyses were performed using RStudio (R Studio Team, 2020) and data are presented as mean ± SD, unless otherwise stated. Analysis of covariance (ANCOVA) was used for all outcomes of interest, to examine the between group (intervention vs. control) differences at follow-up, while controlling for the baseline score (covariate) of that outcome; which is the recommended approach for such experimental designs (Hecksteden et al., 2018; Ritz, 2021). For each comparison, the mean difference and associated 95% confidence interval (CI) are presented. The associated R package to perform this analysis was the “car” package (Fox and Weisberg, 2019). Residual analysis was performed for each model to assess distribution (histograms) and the spread of residuals, as well as homogeneity of regression slopes. The assumptions for each model did not display any extreme deviations from normality, therefore no variables were log transformed. The adjusted mean and 95% CI at follow-up for each group were calculated for all variables [R package “effects” (Fox and Weisberg, 2019)], but the original mean ± SD are also presented. The alpha for determining statistical significance was set at *p* < 0.05.

## Results

### Compliance, training load, and perceived enjoyment

Seven participants attended all 6 training sessions, with one participant attending 5 sessions (due to being absent from school for one training session). An overview of the average external and internal training load (GPS and HR) for each session across the intervention is presented in Table 2. The composite score for perceived enjoyment was similar between session one (median = 70, min = 62, max = 76) and session four (median = 72, min = 59, max = 80) despite the increase in sprints performed. An overview of the individual PACES statement scores can be seen in Table 3.

**Table 2:** Average external and internal training load for each session across the intervention period.

**Session**	**Total distance covered (m)**	**Distance covered per sprint (m)**	**Average top speed (m·s^−1^)**	**Average HR (beats·min^−1^)**	**Average relative exercise intensity (%HRMax)^a^**	**Maximum HR (beats·min^−1^)**	**Maximum relative exercise intensity (%HRMax)^a^**
**6** **× 10 s Sprints**
*1*	280 ± 17 (263–316)	47 ± 3 (44–53)	6.0 ± 0.4 (5.5–6.8)	181 ± 7 (171–191)	88 ± 4 (83–94)	194 ± 5 (186–201)	94 ± 3 (91–100)
*2*	252 ± 21 (215–287)	42 ± 3 (36–48)	5.8 ± 0.3 (5.3–6.4)	179 ± 9 (168–193)	86 ± 3 (82–91)	193 ± 6 (185–203)	93 ± 2 (90–94)
*3^+^*	269 ± 19 (248–300)	45 ± 3 (41–50)	5.8 ± 0.4 (5.3–6.4)	177 ± 8 (163–189)	86 ± 4 (79–88)	192 ± 4 (186–199)	93 ± 3 (89–97)
**8** **× 10 s Sprints**
*4*	337 ± 15 (323–367)	42 ± 2 (40–46)	5.7 ± 0.4 (5.3–6.5)	182 ± 7 (171–191)	88 ± 3 (82–93)	195 ± 5 (185–203)	94 ± 3 (91–99)
*5^++^*	327 ± 41 (242–373)	42 ± 2 (40–47)	5.7 ± 0.5 (5.2–6.5)	179 ± 6 (172–187)	87 ± 3 (82–89)	191 ± 5 (183–197)	92 ± 2 (90–94)
*6*	329 ± 26 (302–376)	42 ± 3 (38–47)	6.3 ± 0.5 (5.6–7.0)	181 ± 9 (167–194)	88 ± 5 (82–96)	194 ± 9 (181–210)	94 ± 4 (90–100)

HR, Heart Rate; HRMax, Maximum Heart Rate.

^a^ Maximum heart rate achieved during the multi-stage fitness test.

^+^n= 5 due to technical issues with GPS units.

^++^n= 7 due to absence from school.

Data are mean ± SD (range).

**Table 3:** Summary of the frequencies for individual statement responses of the PACES questionnaire (*n* = *8*).

	**Session 1 response**					**Session 4 response**				
**Statement**	**Disagree a lot**	**Disagree**	**Unsure**	**Agree**	**Agree a lot**	**Disagree a lot**	**Disagree**	**Unsure**	**Agree**	**Agree a lot**
I enjoy it	0	0	0	4	4	0	0	1	2	5
I feel bored	5	2	0	1	0	5	2	1	0	0
I dislike it	6	2	0	0	0	6	2	0	0	0
I find it pleasurable	0	0	2	5	1	0	0	2	4	2
It's no fun at all	7	1	0	0	0	6	2	0	0	0
It gives me energy	0	0	1	6	1	1	0	1	5	1
It makes me depressed	7	1	0	0	0	7	1	0	0	0
It's very pleasant	0	0	3	3	2	0	0	2	4	2
My body feels good	0	0	2	4	2	0	0	0	4	4
I get something out of it	0	0	1	4	3	0	0	0	3	5
It's very exciting	0	1	2	4	1	0	0	3	2	3
It frustrates me	6	2	0	0	0	6	2	0	0	0
It's not at all interesting	6	1	1	0	0	7	1	0	0	0
It gives me a strong feeling of success	0	0	2	5	1	0	0	1	3	4
It feels good	0	0	2	3	3	0	0	1	3	4
I feel as though I would rather be doing something else	6	2	0	0	0	0	5	3	0	0

Data are shown for session 1 and session 4.

### Differences in physical fitness, risk factors for cardiometabolic disease and cognitive function

Physical fitness and cardiometabolic health outcomes can be found in Table 4, and cognitive function outcomes in Table 5.

**Table 4:** Baseline and follow-up results of physical fitness and cardiometabolic health outcomes, with data presented as mean ± SD.

	**Intervention (*****n*** = **8)**			**Control (*****n*** = **8)**			**Adjusted between-group difference Mean (95% CI)^a^ [*p* value]**
**Outcome**	**Baseline (Mean ±SD)**	**Follow-up** **(Mean ±SD)**	**Adjusted follow-up Mean (95% CI)^a^**	**Baseline** **(Mean ±SD)**	**Follow-up (Mean ±SD)**	**Adjusted follow-up** **Mean (95% CI)^a^**	
MSFT (m)	850 ± 323	1,025 ± 350	999 (884, 1,114)	798 ± 367	873 ± 388	898 (783, 1,013)	100 (−62, 263) [*p* = 0.204]
20 m Sprint (s)	3.77 ± 0.20	3.74 ± 0.25	3.80 (3.70, 3.89)	3.89 ± 0.24	3.65 ± 0.26	3.59 (3.49, 3.69)	0.19 (0.06, 0.33)* [***p*** **=** **0.007]**
Fasting blood glucose (mmol·L^−1^)	4.40 ± 0.36	4.58 ± 0.45	4.68 (4.37, 4.92)	4.60 ± 0.23	4.84 ± 0.32	4.77 (4.49, 5.04)	−0.11 (−0.49, 0.27) [*p =* 0.516]
Fasting plasma insulin (pmol·L^−1^)	59.8 ± 33.9	46.4 ± 21.1	52.9 (43.6, 62.2)	70.0 ± 22.6	49.7 ± 14.0	44.0 (35.4, 52.7)	8.9 (−4.4, 22.1) [*p =* 0.171]
HOMA-IR (AU)	1.96 ± 1.14	1.62 ± 0.81	1.91 (1.52, 2.29)	2.39 ± 0.80	1.79 ± 0.58	1.54 (1.19, 1.90)	0.36 (−0.19, 0.92) [*p =* 0.181]
Blood glucose tAUC (mmol·L^−1^ × 120 min)	726 ± 68	711 ± 115	722 (664, 779)	750 ± 61	728 ± 64	717 (659, 774)	5 (−75, 85) [*p =* 0.898]
Plasma insulin tAUC (pmol·L^−1^ × 120 min)	21,659 ± 6,890	23,685 ± 8,660	28,119 (22,537, 33,702)	32,525 ± 11,059	29,370 ± 10,996	24,935 (19,352, 30,517)	2,279 (−4,931, 9,490) [*p =* 0.434]
IL-6 (pg·ml^−1^)	1.76 ± 1.90	1.02 ± 0.53	1.05 (0.61, 1.48)	1.41 ± 1.38	0.82 ± 0.26	0.85 (0.33, 1.36)	0.20 (−0.49, 0.89) [*p =* 0.526]
IL-10 (pg·ml^−1^)	2.54 ± 0.86	2.55 ± 0.48	2.57 (2.02, 3.11)	3.33 ± 1.72	2.02 ± 0.80	1.95 (1.44, 2.46)	0.62 (−0.14, 1.38) [*p =* 0.102]
IL-15 (pg·ml^−1^)	2.23 ± 0.65	2.14 ± 0.69	2.24 (1.48, 3.00)	2.65 ± 0.61	2.82 ± 1.20	2.71 (1.89, 3.53)	−0.47 (−1.63, 0.69) [*p =* 0.393]
IL-1β (pg·ml^−1^)	45.47 ± 37.20	36.6 ± 16.14	45.79 (29.23, 86.38)	60.83 ± 39.10	52.10 ± 75.93	42.56 (16.84, 83.67)	3.23 (−67.04, 73.49) [*p =* 0.921]

Follow-up and between-group differences adjusted for baseline results are also presented as mean ± 95% CI.

SD, Standard Deviation; CI, Confidence Interval; MSFT, Multi-Stage Fitness Test; HOMA-IR, Homeostatic Model Assessment of Insulin Resistance; tAUC, Total area under the curve; IL, Interleukin.

^a^Adjusted for baseline score.

^*^Statistically significant difference between groups, p < 0.05.

The bolded values are statistically significant results.

**Table 5:** Baseline and follow-up results of cognitive function outcomes, with data presented as mean ± SD.

	**Intervention (*****n*** = **8)**			**Control (*****n*** = **8)**			**Adjusted between-group difference Mean (95% CI) ^a^ [*p* value]**
**Outcome**	**Baseline (Mean ±SD)**	**Follow-up** **(Mean ±SD)**	**Adjusted follow-up Mean (95% CI)^a^**	**Baseline** **(Mean ±SD)**	**Follow-up (Mean ±SD)**	**Adjusted follow-up** **Mean (95% CI)^a^**	
**Response times (ms)**		
Congruent stroop	880 ± 150	778 ± 131	764 (691, 837)	833 ± 143	798 ± 113	811 (738, 884)	−45.7 (−148.3, 56.9) [*p* = 0.347]
Incongruent stroop	1,224 ± 173	1,118 ± 194	1,102 (1,030, 1,175)	1,193 ± 239	1,104 ± 249	1,119 (1,048, 1,191)	−16.5 (−118, 85) [*p =* 0.731]
Sternberg one-item	633 ± 112	629 ± 150	600 (520, 680)	551 ± 53	553 ± 61	583 (502, 663)	14 (−93, 121) [*p =* 0.761]
Sternberg three-item	819 ± 105	766 ± 158	736 (660, 813)	746 ± 86	721 ± 65	751 (675, 827)	−12 (−116, 91) [*p =* 0.784]
Sternberg five-item	974 ± 132	869 ± 152	831 (720, 942)	883 ± 166	882 ± 214	920 (809, 1,031)	−81 (−234, 72) [*p =* 0.254]
Congruent flanker	664 ± 145	580 ± 109	565 (520, 611)	616 ± 128	568 ± 91	583 (537, 628)	−17 (−81, 47) [*p =* 0.576]
Incongruent flanker	705 ± 147	620 ± 138	612 (547, 677)	681 ± 149	633 ± 115	641 (576, 705)	−28 (−120, 64) [*p =* 0.515]
**Accuracy (%)**	
Congruent stroop	97.8 ± 2.5	97.5 ± 3.8	97.7 (95.1, 100.0)	100 ± 0	99.4 ± 3.5	99.2 (96.6, 100.0)	−1.1 (−4.4, 2.2) [*p =* 0.411]
Incongruent stroop	97.6 ± 2.9	95.3 ± 4.1	95.1 (92.8, 97.4)	97.2 ± 2.5	95.9 ± 3.5	96.2 (93.8, 98.5)	−1.0 (−4.0, 2.2) [*p =* 0.499]
Sternberg one-item	98.1 ± 2.9	97.7 ± 3.2	97.6 (95.1, 100.0)	98.4 ± 2.9	97.7 ± 3.2	97.7 (95.1, 100.0)	−0.02 (−3.6, 3.6) [*p =* 0.988]
Sternberg three-item	96.7 ± 2.7	99.6 ± 1.1	99.6 (98.3, 100.0)	97.3 ± 2.0	97.7 ± 2.2	97.6 (96.2, 99.0)	2.0 (0.02, 3.9)* [***p** **=*** **0.046]**
Sternberg five-item	94.7 ± 6.2	96.5 ± 3.5	96.6 (92.7, 100.0)	95.7 ± 6.0	93.8 ± 6.3	93.7 (89.7, 97.6)	2.9 (−2.6, 8.4) [*p =* 0.295]
Congruent flanker	99.6 ± 1.2	99.6 ± 1.2	99.5 (98.6, 100.0)	99.2 ± 1.5	99.6 ± 1.2	99.6 (98.7, 100.0)	−0.1 (−1.4, 1.1) [*p =* 0.855]
Incongruent flanker	99.2 ± 1.54	96.6 ± 3.7	96.6 (93.2, 100.0)	97.5 ± 3.9	96.7 ± 4.7	96.7 (93.2, 100.0)	−0.04 (−4.8, 4.7) [*p =* 0.986]
BDNF (ng·ml^−1^)	18.47 ± 7.51	39.12 ± 9.88	42.9 (37.4, 48.3)	28.69 ± 11.36	22.95 ± 9.13	19.2 (13.8, 24.7)	17.89 (10.7, 25.1)** [***p*** **<** **0.001]**

Follow-up and between-group differences adjusted for baseline results are also presented as mean ± 95% CI.

SD, Standard Deviation; CI, Confidence Interval; BDNF, Brain-Derived Neurotrophic Factor.

^a^Adjusted for baseline score.

^*^Statistically significant difference between groups, p < 0.05.

^**^Statistically significant difference between groups, p < 0.001.

The bolded values are statistically significant results.

For MSFT performance, there was no difference between the intervention and control group at follow-up (*p* = 0.204). For 20 m sprint performance, there was a difference between the intervention and control group at follow-up (*F*_(1, 13)_ = 10.8, *p* = 0.007), whereby the intervention group (3.80 s, 95%CI [3.70–3.89 s]) were slower than the control group (3.59 s, 95%CI [3.49–3.89 s]).

There was no difference between the intervention and control group at follow-up for fasting blood glucose concentration (*p* = 0.516), fasting plasma insulin concentration (*p* = 0.171), HOMA-IR (*p* = 0.181), postprandial blood glucose tAUC (*p* = 0.898) or postprandial plasma insulin tAUC (*p* = 0.434). There was also no difference between the intervention and control group at follow-up for plasma IL-6 (*p* = 0.526), IL-10 (*p* = 0.102), IL-15 (*p* = 0.393) and IL- 1β concentrations (*p* = 0.921).

**Figure 2:**
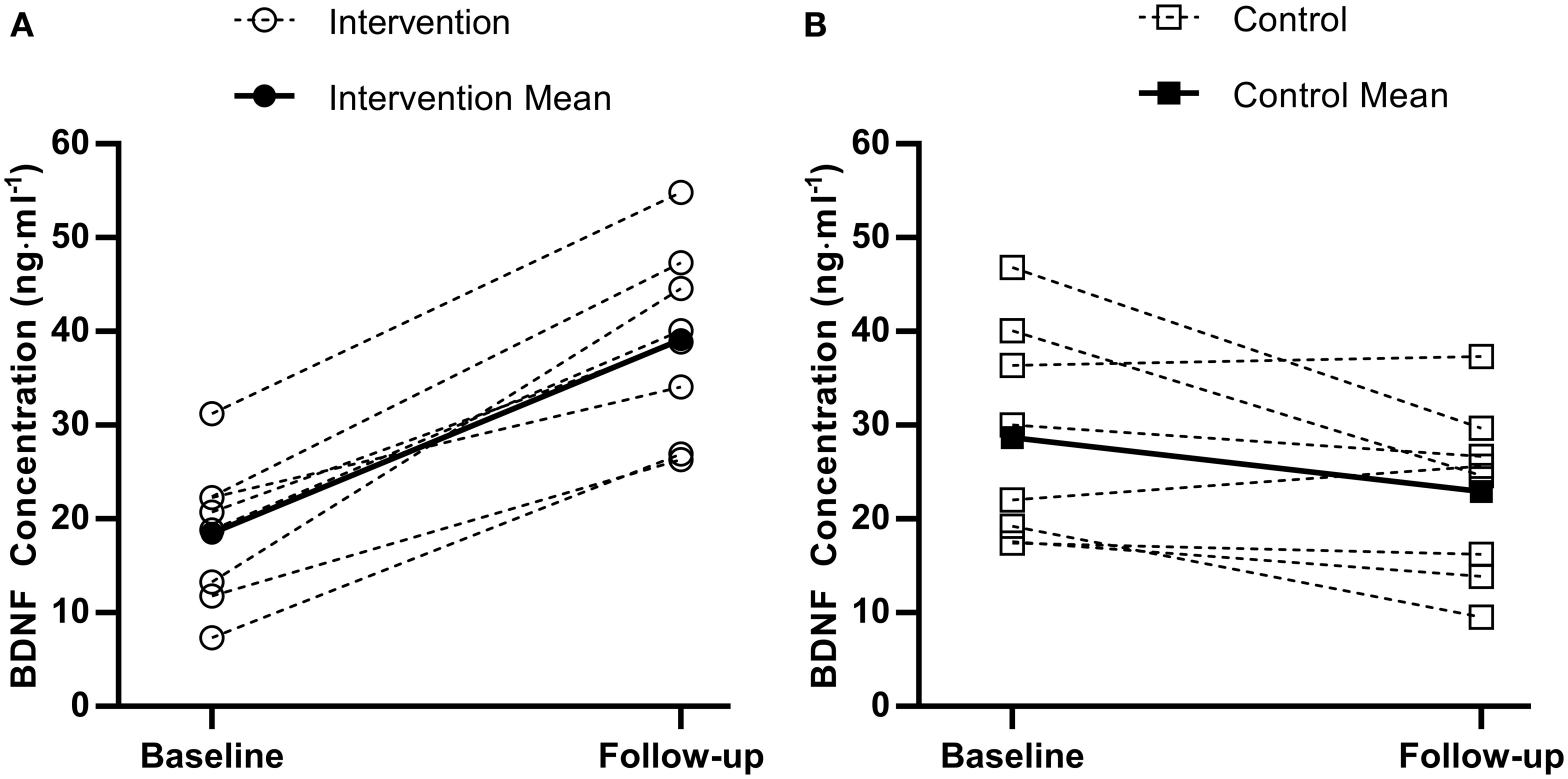
Individual (dashed) and mean (solid) pre to post brain-derived neurotrophic factor (BDNF) changes for the experimental group **(A)** and control group **(B)**.

There was a statistically significant difference between the intervention and control group at follow-up for accuracy during the three-item Sternberg paradigm (*F*_(1, 13)_ = 4.9, *p* = 0.046). Specifically, the intervention group (99.6%, 95%CI [98.3–100%]) were more accurate than the control group (97.6%, 95%CI [96.2–99.0%]). There was no difference between the intervention and control group at follow-up for response times or accuracy on any of the remaining cognitive function tests (all *p* > 0.05). For serum BDNF concentration, there was a difference between the intervention and control group at follow-up [*F*_(1, 13)_ = 38.4, *p* < 0.001, Figure 2], whereby the intervention group (42.9 ng·ml^−1^, 95%CI [37.4–48.3 ng·ml^−1^]) had higher concentrations compared to the control group (19.2 ng·ml^−1^, 95%CI [13.8–24.7 ng·ml^−1^]).

## Discussion

The main findings of the present study are that 2 weeks of a school-based, sprint training intervention improved accuracy during a working memory test and increased BDNF concentrations, but did not affect other aspects of cognitive performance, cardiorespiratory fitness (distance covered on the MSFT) or risk factors of cardiometabolic diseases. However, the intervention group were slower on the 20 m sprint test following the intervention. The present study provides novel contributions to the literature by examining the postprandial response to a standardized breakfast, a range of cytokines related to low-grade inflammation, as well as various domains of cognitive function. Moreover, this is the first study to examine the perceived enjoyment and the effects of an ecologically valid, school-based, sprint-interval intervention in a group of adolescent girls; suggesting that such an intervention is both feasible to run during morning form (before school) and enjoyed by the participants.

The present study is the first to examine the perceived enjoyment of an ecologically valid school-based, low-volume sprint training intervention, in conjunction with the effects of the exercise on cardiometabolic health and cognition. The sprint training intervention was successfully implemented into the school day, without interfering with the curriculum as sessions were held in the morning during form time. This is important, given that it has been previously cited that a lack of time given to PE lessons is one of many reasons for low physical activity during the school day (Boyle et al., 2008; Bond et al., 2017). Thus, such a small inclusion of physical activity throughout the week could potentially lead to the accrual of greater physical activity, though this would need to be examined longer term. Furthermore, a key strength for the feasibility of implementation was that the sessions required minimal set-up time and no specialist equipment, nor would the individual supervising the training require any additional training due to the simplicity of the design. The adolescent girls also reported enjoying the sessions and feeling as though they “got something out of the sessions” and reported favorably upon their perceptions of the intervention (Table 3). Therefore, a key strength of this pilot study is the engagement with the training from the adolescent girls, and the clear feasibility of such sprint-based training interventions.

Two weeks of school-based, sprint training led to greater accuracy (mean difference = 2.0%, 95%CI [0.02–3.9%]) on the three-item level of the Sternberg paradigm (working memory). This is supported by previous work demonstrating that exercise training can lead to less errors on the Wisconsin Sorting Card Test assessing executive function (Chen et al., 2016), as well as general improvements in working memory capacity (Jeon and Ha, 2017). Importantly, this improvement in accuracy was not at the expense of increased response times, which indicates an actual improvement rather than a speed-accuracy trade-off. Although short-term memory ability is suggested to improve throughout adolescence (Ryan, 1990), these data suggest that additional benefits can be achieved through exercise training. This could have important implications, given that working memory is an important domain of cognition related to learning during adolescence and is linked to academic performance (Gathercole et al., 2003).

The present study is the first to demonstrate that a short-term school-based intervention increased resting brain-derived neurotrophic factor (BDNF) concentration in adolescent girls. There are currently limited and equivocal data on resting BDNF concentration following exercise interventions in adolescents (Azevedo et al., 2020). The magnitude of difference in the present study is much larger than that reported after an 8-week treadmill running intervention (Jeon and Ha, 2017), which only reported within-group differences, thus making comparisons difficult. There is some evidence that BDNF increases may be exercise intensity-dependent (Jeon and Ha, 2017), which supports the greater increase observed following sprint training in the present study. Given the sparse data on chronic training and BDNF concentration in adolescents, such a large increase should be interpreted tentatively until further research is conducted. Nonetheless, the present study provides novel evidence that 2 weeks of a school-based sprint training intervention increased BDNF concentration in adolescent girls, alongside a concomitant increase in accuracy on a working memory task.

For the remaining cognitive function outcomes there was no difference between the intervention and control groups after 2 weeks of school-based sprint interval training. It is worth noting that the mean difference in response times suggest the intervention group were quicker, but due to the small sample size these estimates were surrounded by large windows of uncertainty (Table 5). Much of the previous evidence has demonstrated improvements in cognitive function outcomes across a range of domains after a period of exercise training in adolescents (Schmidt et al., 2015; Chen et al., 2016; Bond et al., 2017; Jeon and Ha, 2017; Ludyga et al., 2018). These interventions were generally performed for a longer duration (6 to 12 weeks), more frequently (from two to five sessions per week) and at a lower intensity (ranging from 60 to 70% HR_max_ and 40–70% V°O_2max_) than the present study. Collectively, these results suggest that cognitive function will benefit from a higher volume and longer duration exercise intervention of a moderate intensity in adolescents. However, the present study, to the authors' knowledge, is the first examining the effects of short-term high-intensity exercise interventions on cognitive function in adolescents. To provide a more robust evidence base on the efficacy of short-term interventions, more work is needed, particularly to explore the manipulation of training volume and intensity over a short period.

Data from the present study demonstrate that cytokine concentrations (IL-6, IL-10, IL-15 and IL-1β) were not affected by the two-week sprint training intervention. These findings are similar to those of Buchan et al. (2013), whereby an 8-week sprint training intervention did not elicit reductions in IL-6 concentration in older adolescents. Interestingly, these data collectively oppose the evidence in adults that regular physical activity is negatively associated with IL-6 concentration (Pedersen and Febbraio, 2008) and exercise interventions reduce basal IL-6 concentration (Di Raimondo et al., 2016; Cronin et al., 2017). IL-6 is a pleiotropic cytokine, secreted from several sources and is involved in many physiological processes (Cronin et al., 2017; Hoffmann and Weigert, 2017). Therefore, the exact origin of the systemic IL-6 concentrations might explain the abundance, or lack thereof, in response to exercise interventions (Di Raimondo et al., 2016). Nonetheless, this is the first study to examine the effects of school-based sprint-training on markers of inflammation in adolescent girls; who may be prone to higher basal concentrations (Williams et al., 2022). Future research should further examine different exercise interventions (manipulating exercise intensity, volume, and frequency) and the effects on low-grade chronic inflammation in adolescent girls.

In the present study, 2 weeks of school-based sprint training did not improve fasting markers of metabolic health (fasting glucose, insulin and homeostatic model assessment of insulin resistance [HOMA-IR]) in adolescent girls. These data, collectively with previous findings (Buchan et al., 2013; Bond et al., 2015; Logan et al., 2016; Cockcroft et al., 2019; Martin-Smith et al., 2019), suggest that exercise interventions generally do not improve markers of fasting metabolic health, in healthy adolescents. Additionally, the postprandial glycaemic and insulinaemic responses to a mixed meal were not affected by 2 weeks of school-based sprint training. To the authors' knowledge, there are only two previous investigations examining the postprandial response to a test meal following an exercise intervention in healthy adolescents (Bond et al., 2015; Cockcroft et al., 2019), whereby responses were not affected by the interventions, in line with the findings of the present study. There is evidence that postprandial glycaemic and insulinaemic responses to an oral glucose tolerance test (OGTT) are affected by exercise training in overweight/obese adolescents (Tjønna et al., 2009). Overall, it seems that exercise interventions may be more effective at improving metabolic health in those who have greater scope for improvement (i.e., overweight/obese), which might provide scientific rationale for school-based interventions to be designed for vulnerable adolescents, including those classified as overweight/obese.

Participants in the intervention group were slower on the 20 m sprint at follow-up compared to the control group (adjusted mean difference = 0.19 s, 95%CI [0.06–0.33 s]). Due to the re-scheduling of follow-up data collection sessions (due to COVID-19 school closures), there were no indoor facilities available to perform the fitness testing. Therefore, the MSFT and 20 m sprint at follow-up were performed on an outdoor court. Average wind speed during these tests was 2.24 m·s^−1^ (5 mph) for the control group and 8.49 m·s^−1^ (19 mph) for the intervention group, but the relative humidity (control; 62% vs. intervention; 60%) and ambient temperature (control; 7°C vs. intervention; 6°C) were similar. Indeed, the greater wind speed experienced by the intervention group during the sprints likely explains their poorer performance.

The present study also has limitations that need acknowledging. Firstly, the small sample size is a clear limitation—especially with the expected small effects in outcome variables from such a short-duration, low volume training intervention. Nonetheless, the intervention was successfully implemented during morning form (before school)—rather than being embedded into physical education lessons, allowing for additional physical activity provision (Bond et al., 2017). Based on this, and the perceived enjoyment of the sessions, the protocol can be altered to provide a slightly higher exercise volume for future investigation. Furthermore, it should be acknowledged that there may be an element of self-selection with training studies. Indeed, with regard to performance on the MSFT the sample of the present study were in the 80^th^-90^th^ percentiles, in reference to Tomkinson et al. (2016). Nonetheless, gift vouchers (Amazon) were offered to all participants that completed the study to try and alleviate this. Though it may also be attributable to the local area, which ranks in the 10^th^ decile on the Index of Multiple Deprivation (least deprived decile). Another limitation of the present study is the lack of habitual physical activity assessment which would help to characterize participants' activity levels, but also provide a useful outcome measure. This should also be included in future work.

Overall, the present study reports upon an enjoyable school-based sprint training intervention, which following a 2-week implementation enhanced the accuracy of working memory and increased BDNF concentration; whilst there was no effect on other aspects of cognitive function or risk factors for cardiometabolic disease. Importantly, this intervention required a minimal time commitment (no longer than 10 mins) and was performed before the start of school lessons. Future research should examine the longer-term effects of such a sprint training intervention within a school setting, and the subsequent effects on cardiometabolic health and cognitive function that occur following a more prolonged period of training.

## Data availability statement

The raw data supporting the conclusions of this article will be made available by the authors, without undue reservation.

## Ethics statement

The studies involving human participants were reviewed and approved by Nottingham Trent University Human Ethics Committee. Written informed consent to participate in this study was provided by the participants' legal guardian/next of kin.

## Author contributions

RW, SC, JM, and MN: design of the study. RW, SC, KD, and CS: data collection. RW, KD, JM, and SC: data analysis. RW, SC, KD, CS, and MN: writing—reviewing and editing. All authors read and approved the final version of the manuscript.

## Conflict of interest

The authors declare that the research was conducted in the absence of any commercial or financial relationships that could be construed as a potential conflict of interest.

## Publisher's note

All claims expressed in this article are solely those of the authors and do not necessarily represent those of their affiliated organizations, or those of the publisher, the editors and the reviewers. Any product that may be evaluated in this article, or claim that may be made by its manufacturer, is not guaranteed or endorsed by the publisher.
